# A case of necrotizing fasciitis initially misdiagnosed as cellulitis

**DOI:** 10.1016/j.ijscr.2024.109701

**Published:** 2024-04-24

**Authors:** Aditya Joshi, Talal Alomar, Diego F. Kaune, Julien Bourgeois, David Solomon

**Affiliations:** a Creighton University School of Medicine, Phoenix, AZ, United States of America; b Johns Hopkins University, Baltimore, MD, United States of America; c Phoenix Children's Hospital Department of Hospital Medicine, Phoenix, AZ, United States of America

**Keywords:** Necrotizing fasciitis, Cellulitis, Surgical debridement, Necrotizing soft tissue infection

## Abstract

**Introduction and importance:**

Necrotizing Fasciitis (NF) is a life-threatening, rapidly progressive infection of the skin and underlying soft tissues. Bacterial pathogens induce a toxic-shock reaction that reduces vascular flow, causing thrombosis, sepsis, and tissue necrosis. Treatment consists of immediate IV antibiotics and oftentimes surgical intervention. We present a case of acute NF that was misdiagnosed as cellulitis.

**Case presentation:**

A 17-year-old male was transferred to an emergency department from a rural hospital for further management of right lower extremity cellulitis and suspected sepsis. On examination, there was an ulcerated lesion on his right lower leg. Within 4 h, the patient underwent fasciotomy and debridement. The patient was hospitalized for 10 days, received a 3-week-course of Cefazolin, and underwent a meshed split-thickness skin graft. By the end of his hospital stay, he showed significant clinical improvement.

**Clinical discussion:**

Misdiagnosis of NF will almost always lead to a poorer prognosis. The Laboratory Risk Indicator for Necrotizing Fasciitis (LRINEC) score is used to differentiate NF from other soft tissue infections. Yet, other diagnostic clues such as presentation or pain out of proportion to physical findings may be more relevant clinical indicators for a NF diagnosis. Moreover, though imaging findings of NF may be relevant, surgical fascial examination must not be delayed for the purpose of imaging. It is also important to note that cellulitis and NF do share a disease spectrum.

**Conclusion:**

A life-threatening NF infection may seem to be a benign-appearing case of cellulitis, and thus early detection is vital.

## Highlights

• Necrotizing fasciitis misdiagnosed as cellulitis can lead to delayed intervention.
• Unlike cellulitis, NF progresses rapidly with disproportionate pain.
• Imaging and diagnostic clues are useful; the gold standard is surgical examination.
• NF treatment requires immediate antibiotics, debridement, and reconstruction.
• In early-stage NF, diagnostic distinction from cellulitis may be difficult.

## 1 Introduction

Necrotizing Fasciitis (NF) is a rapidly progressive necrotizing soft-tissue infection that travels along avascular planes, commonly including muscle fascia and overlying subcutaneous fat [1]. Compared to cellulitis, a more superficial infection of the vascular dermis and subcutaneous fat, limited vascular supply in the fascia inhibits immune response and slows leukocyte migration, allowing bacterial pathogens to proliferate rapidly [2]. Untreated NF leads to tissue necrosis and may progress to toxic shock, which is associated with high rates of morbidity and mortality.

The initial presentation of soft tissue infections is characterized by localized swelling, erythema, and pain, often present in both cellulitis and NF [3,4]. While antibiotic therapy is the treatment of choice for all soft tissue infections, patients with lesions concerning for necrotizing fasciitis must undergo urgent surgical intervention in addition to enhanced-spectrum antibiotics, as common pathogens include group A Streptococcus, *Staphylococcus Aureus*, Clostridium, and mixed gram-negative and anaerobic organisms. [5]

In this report, we present a case of NF that was initially diagnosed as cellulitis and discuss current differential building, workup, and rule-out protocols for NF in the acute care setting.

## 2 Case presentation

**Fig. 1:**
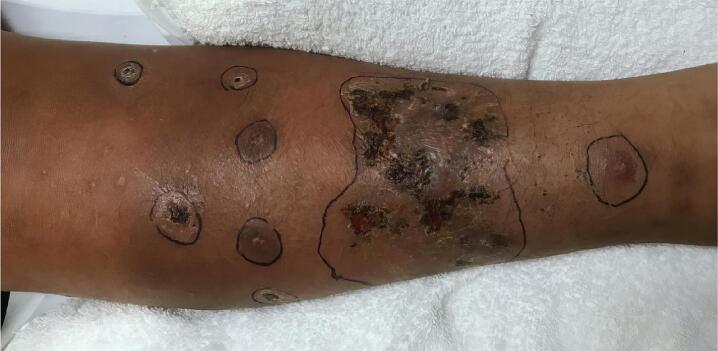
Initial presentation of patient in emergency department. Multiple ulcerated, coalescing, violaceous, and necrotic lesions with surrounding erythema on the right lower leg.

A 17-year-old male with a history of obesity and well-controlled asthma was transferred to an emergency department from a rural hospital for further management of sepsis secondary to right lower extremity cellulitis. Three days prior, he had noticed an itchy scratch on his right shin which had progressively become swollen, erythematous, and tender. The patient denied any paresthesia or numbness but had developed a limp due to the pain. The pain was localized at the anterior lesion but extended to the lateral and posterior surfaces of the calf. The patient reported feeling feverish alongside a headache without vision changes, neck stiffness, or photophobia/phonophobia. The patient had two cats and two dogs at home but denied any scratches or bites, as well as any tick or insect bites. He also denied vomiting, diarrhea, abdominal pain, nausea, rash, malaise, arthralgias, prior MRSA infection, or sick contacts. The patient's only recent travel was to Arizona from his home in Nebraska (Fig. 1).

At the outside hospital, the patient had been afebrile, mildly tachycardic (110 bpm), and tachypneic (22 bpm), with an oxygen saturation of 90 % on room air. Laboratory results were notable for elevated white blood cell count (26.7 × 10^9/L) with neutrophilic predominance (89.9 %), elevated CRP of 33 mg/dL, and elevated lactate of 2.2 mmol/L. The patient also had an elevated ALT of 93 U/L. CT with contrast of the lower extremity showed subcutaneous edema of superficial soft tissue, along with normal-appearing muscle and fascia consistent with cellulitis. He received 4 L normal saline, 1× vancomycin sepsis dose, 6000 mg cefazolin IV, 200 mg doxycycline IV.

**Fig. 2:**
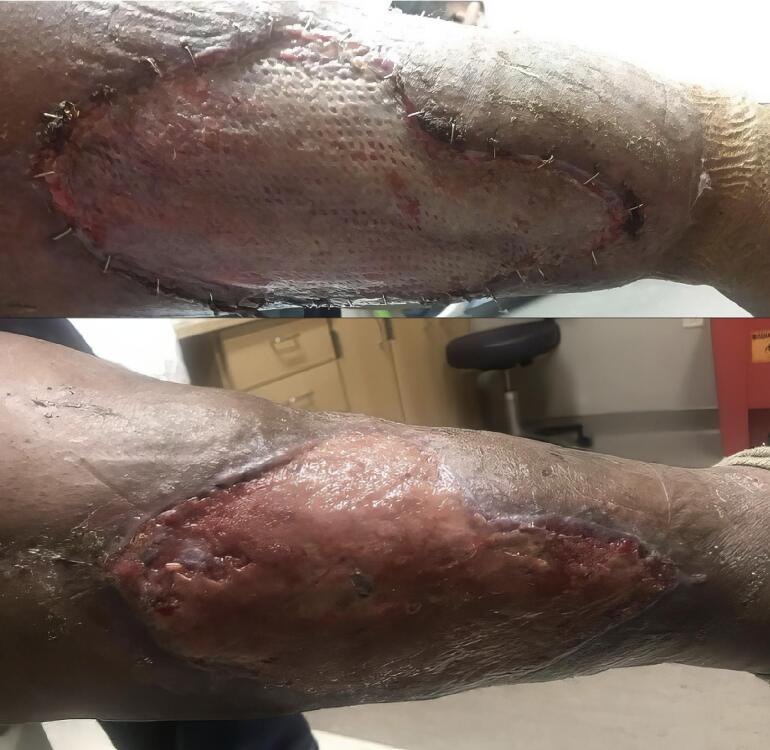
Top: Wound after debridement and skin graft placement; bottom: Patient's leg one month after skin graft.

On arrival to the emergency department, the patient was afebrile, mildly tachycardic (102 bpm), and tachypneic (24 bpm), with an oxygen saturation of 94 % on room air. On examination, there was an 8x6cm coalescing, necrotic, ulcerated lesion with a violaceous base on his right lower leg. Surrounding the central lesion were 1 cm fluctuant lesions, some with hemorrhagic crust and central necrosis. The lower leg was diffusely tender and erythematous (Fig. 2).

A second read of his prior CT scan also concluded subcutaneous edema consistent with cellulitis. The patient was continued on doxycycline and cefazolin and admitted to the floor. General surgery was consulted for further evaluation and agreed with the diagnosis of cellulitis.

**Fig. 3:**
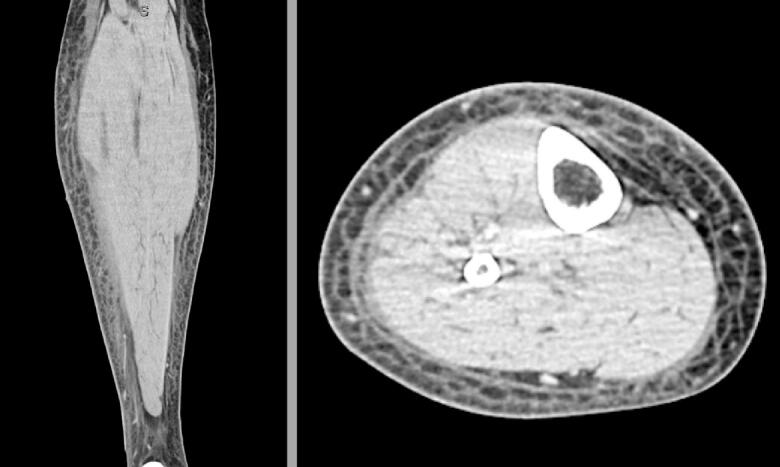
Coronal and axial view of CT scan of patients right lower extremity. First read at outside hospital was remarkable for subcutaneous edema consistent with cellulitis. Second opinion read at second hospital concluded cellulitis with no evidence of abscess or bone/joint abnormality.

Within a few hours, the patient began reporting extreme pain. Due to the severity of the pain and the rapid progression of the disease course, plastic surgery was urgently consulted for concern of necrotizing infection. Within 4 h, the patient underwent fasciotomy and debridement. The following day, the debridement was repeated, and a meshed split-thickness skin graft taken from the patient's thigh was secured. Wound cultures grew multiple species of Group A *Streptococcus* as well as *Staphylococcus*. The patient was hospitalized for 10 days and received a 3-week course of Cefazolin (Fig. 3).

It is relevant to note that the work has been reported in line with SCARE criteria [6].

## 3 Discussion

In NF, patients develop necrosis of soft tissue within hours to days, which can quickly progress to irreversible limb damage, sepsis, and death. With a high mortality rate of up to 24–34 % and a 22 % amputation rate once diagnosed, swift surgical and medical intervention is imperative [7].

As in the case of our patient, prior literature reports that NF is misdiagnosed in 70–85 % of cases, often due to symptoms resembling cellulitis and other soft tissue infections [8]. Unlike cellulitis, NF tends to progress rapidly with severe and disproportionate pain, systemic signs such as fever, and subcutaneous crepitus that may be detected upon palpation [1]. Regardless, the misdiagnosis of NF will almost always lead to delayed intervention and a poorer prognosis. In patients diagnosed with cellulitis with acute worsening or classic warning signs, physicians should have a low threshold for further evaluation, IV antibiotics, and surgical intervention.

The Laboratory Risk Indicator for Necrotizing Fasciitis (LRINEC) score, developed by Wong et al. in 2004, is used to stratify soft tissue infections as high risk (≥8), moderate risk (6–7), and low risk (≤5) for necrotizing fasciitis [9]. However, waiting for lab results may be impractical in acute settings, and current recommendations are operative management if history and physical exam are concerning for NF. It is relevant to note that our patient had a LRINEC score of 6 (moderate risk).

While Wong et al. showed the LRINEC score to have a positive predictive value of 92.0 % and a negative predictive value of 96.0 %, subsequent data has shown it to have limited sensitivity [10]. Thus, other diagnostic clues such as serum lactate levels above 2.0 mmol/L at presentation [11] or pain out of proportion to physical findings [10] may, at times, be more relevant clinical indicators.

Imaging findings of NF may include asymmetric fascial thickening, soft-tissue air, blurring of fascial planes, inflammatory fat stranding, reactive lymphadenopathy, and nonenhancement of the muscular fascia [12]. In this case, the subcutaneous edema noted on CT had most likely progressed significantly by the time of transfer, and operative intervention was performed before repeat imaging was obtained. Nevertheless, the gold standard for the diagnosis of NF remains direct surgical fascial examination, and surgery must not be delayed for imaging [4]. Therein, history and physical exam remain the most rapid assessments for urgent intervention.

Once suspected, NF necessitates immediate and aggressive intervention. Treatment includes hemodynamic support, broad-spectrum antibiotics, and surgical debridement to remove necrotic tissue and control the spread of the infection along fascial planes. Often, close monitoring and repeat debridement is required, with reconstructive options viable only after source control has been obtained. [13] Some retrospective studies, case-controlled studies, and small randomized trials have also suggested intravenous immune globulin (IVIg) therapy as an additional option in the treatment of NF [14].

In early-stage fasciitis, diagnostic distinction from cellulitis may be difficult. Thus, clinicians must remain vigilant for NF in the initial workup of localized soft tissue infection.

## Declaration of competing interest

The authors declare that they have no known competing financial interests or personal relationships that could have appeared to influence the work reported in this paper.
